# Association between abnormal lipid profile and inflammation and progression of myelodysplastic syndrome to acute leukemia

**DOI:** 10.1186/s40164-022-00309-7

**Published:** 2022-09-16

**Authors:** Wei Qiao, Elliana Young, Chun Feng, Suyu Liu, Jeff Jin, Laila Noor, Cristhiam M. Rojas Hernandez, Gautam Borthakur, Olga Gorlova, Vahid Afshar-Kharghan

**Affiliations:** 1 Department of Biostatistics, The University of Texas MD Anderson Cancer Center, 6565 MD Anderson Blvd., Suite Z9.5044, Houston, TX 77030 USA; 2 Information Services, Enterprise Development & Integration, The University of Texas MD Anderson Cancer Center, 6565 MD Anderson Blvd., Suite Z9.5044, Houston, TX 77030 USA; 3 Pharmacy Quality-Regulatory, The University of Texas MD Anderson Cancer Center, 6565 MD Anderson Blvd., Suite Z9.5044, Houston, TX 77030 USA; 4 Department of Pulmonary Medicine, The University of Texas MD Anderson Cancer Center, 6565 MD Anderson Blvd., Suite Z9.5044, Houston, TX USA; 5 Section of Benign Hematology, The University of Texas MD Anderson Cancer Center, 6565 MD Anderson Blvd., Suite Z9.5044, Houston, TX 77030 USA; 6 Department of Leukemia, The University of Texas MD Anderson Cancer Center, 6565 MD Anderson Blvd., Suite Z9.5044, Houston, TX 77030 USA; 7 Section of Epidemiology and Population Science, Department of Medicine, Baylor College of Medicine, 6565 MD Anderson Blvd., Suite Z9.5044, Houston, TX 77030 USA

**Keywords:** Myelodysplastic syndrome, Acute Myeloid Leukemia, Clonal hematopoiesis, Inflammation, Triglyceride

## Abstract

Clonal hematopoiesis of indeterminate potential (CHIP) is associated with a small risk of developing hematologic malignancies and a higher risk of cardiovascular diseases (CVD). We asked whether the reverse correlation exists and cardiometabolic risk factors have an impact on the progression of myelodysplastic syndrome (MDS) to acute myeloid leukemia (AML). We investigated the association between abnormal lipid profiles and inflammation in MDS, which shares many genetic mutations with CHIP, and the risk of developing acute leukemia. We examined the medical records of 11071 MDS patients. Among them, 5422 had at least one lipid profile or C-reactive protein (CRP) measurement. In univariate and multivariate analyses, elevated triglyceride and high-sensitive C-reactive protein (HS-CRP) were significantly associated with a diagnosis of acute leukemia in MDS patients. Next, we examined these associations in patients with available MDS prognostic scores (International Prognostic Scoring System, IPSS, or its revised version IPSS/R) (n = 2786 patients). We found that the statistical association between CRP and the progression of MDS to leukemia was independent of other variables in the scoring system. MDS patients with elevated CRP in both the high-risk and low-risk groups had a higher risk of progression to AML than those with a lower CRP. We speculate that inflammation might be a common denominator in developing hematologic malignancies and CVD in patients with clonal hematopoiesis.

**Supplementary Information:**

The online version contains supplementary material available at 10.1186/s40164-022-00309-7.

## Keypoints

Elevated triglyceride and high-sensitive CRP are associated with a higher risk of acute leukemia in MDS.

**Supplementary Information:**

The online version contains supplementary material available at 10.1186/s40164-022-00309-7.

To the editor:

 MDS is a premalignant blood disorder, and about 30% of MDS patients develop AML. The presence of similar genetic mutations in abnormal hematopoietic clones in CHIP and MDS raises the possibility of a CHIP to MDS to AML continuum [1–6]. CHIP is also associated with a significant risk of CVD [7]. Preventive measures such as the treatment of hyperlipidemia, blood pressure, and diabetes are recommended in managing CHIP patients [8]. Whether cardiovascular risk factors impact the progression of CHIP to malignant blood disorders is unknown. MDS is also a clonal blood disorder but with a much higher conversion rate to AML than CHIP. We investigated the association between abnormal lipid profile and elevated inflammation biomarkers and the frequency of diagnosis of AML in a large MDS cohort.

Among 11071 MDS patients diagnosed and treated between 2003 and 2020 at MD Anderson Cancer Center, 5422 had at least one measurement of their lipid profile, CRP, or HS-CRP. Their average age was 56 (+ 12), and 59.4% were male. More information about the population and laboratory results are available in Additional file 1: Methods and Table S1. About 62.2% (n = 3375) of MDS patients were diagnosed with acute leukemia through follow-up. We compared laboratory values between MDS patients who developed AML and those who remained leukemia-free (n = 2047, 37.8%). Among the continuous variables, triglyceride and VLDL were higher, and HDL and LDL were lower in MDS patients who developed acute leukemia than in those who did not (Additional file 1: Table S2). In the univariate analysis, all the continuous lab variables, as well as the statin prescription, were significantly associated with the leukemia status (Table 1). In the multivariate analysis, only the triglyceride (OR 1.44; 95% CI 1.16–1.78) and HS-CRP (OR 1.13; 95% CI 1.06–1.21) remained significantly associated with a diagnosis of acute leukemia (Table 1). In the univariate analysis, treatment with statins was associated with an increased frequency of diagnosis of AML (OR 1.13, 95% CI 1.00–1.28, p = 0.044) (Table 1). However, one cannot study the effect of statins without considering the possibility of confounding effects of high LDL. In the multivariable analysis, when forced into the model, statins were associated with a modestly reduced risk of AML, with a borderline statistical significance (OR 0.8, CI 0.63–1.01, p = 0.061) (Table 1).

**Table 1:** Risk of leukemia for all patients

	Univariate logistic regression		Multivariable logistic regression (based on backward model selection)		Multivariable Model (based on a backward model selection with statin forced in the Model)	
	OR (95% CI)	p-value	OR (95% CI)	p-value	OR (95% CI)	p-value
HS-CRP*	1.18 (1.11, 1.24)	< 0.001	1.13 (1.06,1.21)	< 0.001	1.14 (1.06,1.21)	< 0.001
TRIG*	1.38 (1.22, 1.56)	< 0.001	1.44 (1.16,1.78)	0.001	1.44 (1.16,1.78)	0.001
CRP*	1.28 (1.20, 1.37)	< 0.001				
HDL	0.99 (0.98, 0.99)	< 0.001				
LDL	1.00 (0.99, 1.00)	0.001				
VLDL	1.01 (1.00, 1.01)	< 0.001				
Statin, yes/no	1.13 (1.00, 1.28)	0.044			0.80 (0.63,1.01)	0.061

^*^Log-transformed, OR > 1 means an increased risk

Using IPSS or IPSS/R classifications (whichever was available initially), MDS patients were categorized into two groups: patients with a high risk of conversion to leukemia (high and intermediate II according to IPSS and high and very high according to IPSS/R) and those with a low risk (Intermediate I and low according to IPSS and low and very low according to IPSS/R). In our cohort, 2786 patients had either IPSS or IPSS/R and lipid profile and CRP or HS-CRP. Among them, 1003 (36%) were in the high-risk group and 1783 (64%) in the low-risk group. The distribution of lipid profile and CRP and HS-CRP among these groups are shown in Additional file 1: Table S3. In the low-risk group, elevated CRP was more common in patients who developed leukemia than in those who did not (Additional file 1: Table S4). In the high-risk group, elevated CRP and triglyceride were more common in patients who developed leukemia (Additional file 1: Table S5). Elevated CRP was associated with an increased risk of conversion to AML in both high- and low-risk groups in univariate and multivariable logistic regression analyses (an estimated odd ratio of 1.3) (Table 2). Our results point to a predictive value for inflammatory biomarkers in MDS. In another study on a small number of low-risk MDS patients, CRP was found to be associated with a worse prognosis [9]. In univariate analysis, the use of statins was associated with an increase in the risk of AML but lost its significance in the multivariable analysis (Table 2). More information about the statistical methods is provided in the Additional file 1: Material.

**Table 2:**
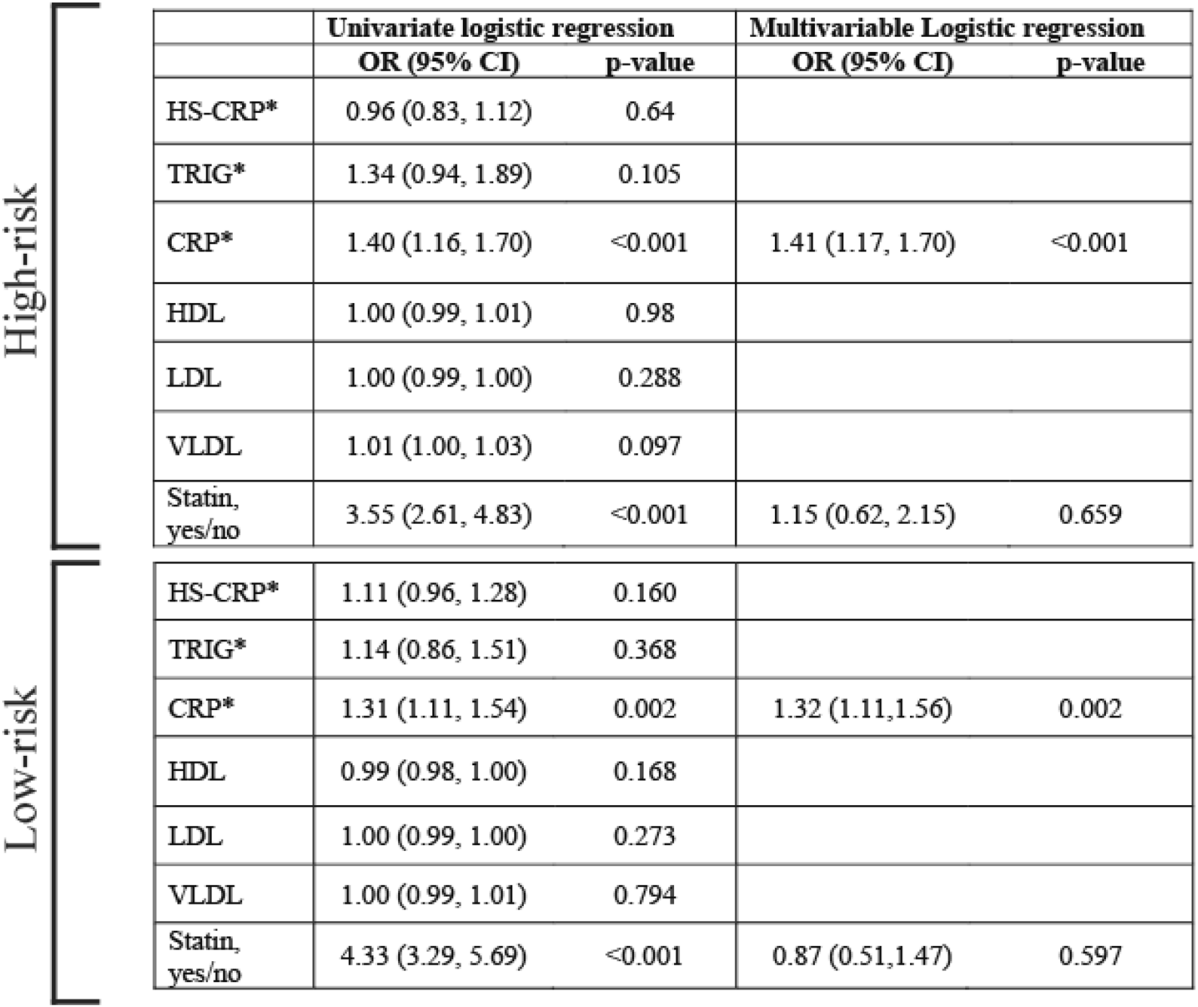
Impact on the risk of leukemia in the high- and low-risk groups

Inflammation is important in the pathogenesis and progression of MDS [10, 11]. The pro-inflammatory effect of elevated triglycerides in atherosclerosis has been recently recognized [12] and might also be relevant to its impact on the bone marrow microenvironment. A limitation of our study is that we did not examine confounding factors such as age, gender, and chemotherapy protocols. Our results and speculations should be examined in other MDS cohorts.

## Supplementary Information

**Additional file 1: Table S1.** Characteristics of the laboratory tests of the patients. **Table S2.** Lab test characteristics stratified by the leukemia status. **Table S3.** Distribution of Lab values in risk subgroups. **Table S4.** Lab test characteristics stratified by the leukemia status for the low-risk group (n=1783). **Table S5.** Lab test characteristics stratified by the leukemia status for the high-risk group (n=1003)

## Data Availability

Data generated or analyzed during this study are included in this published article [and its supplementary information files]. Additional raw data are available from the corresponding author on reasonable request and upon approval of the Institutional Review Boards.
