# Supplementary material for: Association between abnormal lipid profile and inflammation and progression of myelodysplastic syndrome to acute leukemia
Source: Exp Hematol Oncol. 2022 Sep 16;11:58. doi: 10.1186/s40164-022-00309-7 (PMC9479397; doi:10.1186/s40164-022-00309-7)
Supplement: Supplementary file 1 — Additional file 1: Table S1. Characteristics of the laboratory tests of the patients. Table S2. Lab test characteristics stratified by the leukemia status. Table S3. Distribution of Lab values in risk subgroups. Table S4. Lab test characteristics stratified by the leukemia status for the low-risk group (n=1783). Table S5. Lab test characteristics stratified by the leukemia status for the high-risk group (n=1003) [file 40164_2022_309_MOESM1_ESM.docx]

**Supplemental Material**

**Methods**

All of the studies conducted in this manuscript were approved by the Institutional Review Board of the University of Texas M. D. Anderson Cancer Center.

Population

We used ICD diagnostic codes (ICD9 and ICD10 codes) to interrogate the MD Anderson Enterprise Institution data Warehouse and identified 11071 patients with a diagnosis of MDS between 2003 and 2020. Among them, 5898 patients received one of the diagnostic codes for acute myeloid leukemia. We used the same database to extract the results for LDL, HDL, VLDL, CRP, and HS-CRP laboratory tests in MDS patients and identified 5422 MDS patients with at least one of these tests. In addition, we used the MD Anderson pharmacy database to identify 2083 MDS patients (18.8% of total MDS patients) with one of the statins ([Atorvastatin](https://www.uptodate.com/contents/atorvastatin-drug-information?search=statins&source=panel_search_result&selectedTitle=1%7E132&usage_type=panel&display_rank=1), [Lovastatin](https://www.uptodate.com/contents/lovastatin-drug-information?search=statins&source=panel_search_result&selectedTitle=1%7E132&usage_type=panel&display_rank=1), [Simvastatin](https://www.uptodate.com/contents/simvastatin-drug-information?search=statins&source=panel_search_result&selectedTitle=1%7E132&usage_type=panel&display_rank=1), [Fluvastatin](https://www.uptodate.com/contents/fluvastatin-drug-information?search=statins&source=panel_search_result&selectedTitle=1%7E132&usage_type=panel&display_rank=1), [Pitavastatin](https://www.uptodate.com/contents/pitavastatin-drug-information?search=statins&source=panel_search_result&selectedTitle=1%7E132&usage_type=panel&display_rank=1), [Pravastatin](https://www.uptodate.com/contents/pravastatin-drug-information?search=statins&source=panel_search_result&selectedTitle=1%7E132&usage_type=panel&display_rank=1), or [Rosuvastatin](https://www.uptodate.com/contents/rosuvastatin-drug-information?search=statins&source=panel_search_result&selectedTitle=1%7E132&usage_type=panel&display_rank=1)) listed in their medication/pharmacy profile.

We examined the association between lipid profile and inflammatory biomarkers and established prognostic factors for MDS, International Prognostic Scoring System (IPSS and revised IPSS or IPSS/R). These scoring systems consider several variables such as cytopenia, karyotypes, and the number of blasts in the bone marrow and are used to predict progress to acute leukemia and survival of patients with MDS. We identified 2786 MDS patients who had IPSS or IPSS/R scores, lipid profiles, and CRP or HS-CRP. According to IPSS and IPSS/R scores, patients were classified into high- (including high and very high risk, and intermediate II) and low-risk (low and very low-risk, and intermediate I) categories.

Statistical analysis

We used the averaged results for subjects with more than one measurement of the same variable. The distribution of each continuous variable was summarized by its mean, standard deviation, and interquartile range. The distribution of each categorical variable was summarized in terms of its frequencies and percentages. Continuous variables were compared between groups by a Wilcoxon rank-sum test. Fisher’s Exact test examined the associations between categorical variables and groups. Univariate and multivariable logistic models were conducted, with the lab test results treated as predictors and the leukemia status as the outcome. The variables with p≤0.05 in the univariate analyses were considered in the multivariable model. Backward model selection was used when all patients were considered. The backward model selection removes variables from the model until all remaining variables are statistically significant. At the same time, statin treatment (yes vs. no) was forced in the final multivariable model regardless of significance due to clinical importance. For subgroup analysis, that is, high-risk or low-risk patients based on IPSS scores, the variables with p≤0.05 in the univariate analyses were kept in the multivariable models. All computations were carried out in SAS version 9.4.

**Supplementary Tables**

| **Supplementary Table 1**. Characteristics of the laboratory tests of the patients | | | | | | |
| --- | --- | --- | --- | --- | --- | --- |
| Variable | N | Mean | Std Dev | Median | Minimum | Maximum |
| \| C-reactive protein (CRP)* \| \| --- \| \| High-sensitivity CRP (HS-CRP)* \| \| Average high-density lipoprotein (HDL) \| \| Average low-density lipoprotein (LDL) \| \| Average triglycerides (TRIG)* \| \| Average very low-density lipoprotein (VLDL) \| | \| 1444 \| \| --- \| \| 2149 \| \| 3234 \| \| 2673 \| \| 4044 \| \| 2880 \| | \| 60.56* \| \| --- \| \| 38.45* \| \| 42.56 \| \| 87.22 \| \| 174.56* \| \| 31.00 \| | \| 68.62 \| \| --- \| \| 60.14 \| \| 17.30 \| \| 36.33 \| \| 109.64 \| \| 14.75 \| | \| 35.46 \| \| --- \| \| 11.50 \| \| 40.00 \| \| 84.00 \| \| 149.02 \| \| 28.00 \| | \| 0.17 \| \| --- \| \| 0.21 \| \| 4.50 \| \| 0.00 \| \| 21.00 \| \| 6.00 \| | \| 450.00 \| \| --- \| \| 508.00 \| \| 187.00 \| \| 344.00 \| \| 1833.69 \| \| 138.00 \| |

*A highly skewed distribution

**Supplementary Table 2**: Lab test characteristics stratified by the leukemia status

| Variable | Leukemia | N | Mean ± SD, median (interquartile) | Wilcoxon Rank sum test p-value |
| --- | --- | --- | --- | --- |
| HDL (mg/dL) | N | 1260 | 44.58 ± 17.81, 42 (32.53, 54) | <0.001 |
|  | Y | 1974 | 41.27 ± 16.84, 39 (30, 50.5) |  |
| LDL (mg/dL) | N | 1083 | 90.03 ± 36.5, 87.5 (63, 113) | 0.001 |
|  | Y | 1590 | 85.3 ± 36.1, 81.06 (59, 108.5) |  |
| TRIG (mg/dL) | N | 1511 | 164.71 ± 103.56, 140 (99.5, 200.5) | <0.001 |
|  | Y | 2533 | 180.43 ± 112.72, 155.7 (109.5, 221.5) |  |
| VLDL (mg/dL) | N | 1125 | 29.75 ± 15.05, 26 (19, 38) | <0.001 |
|  | Y | 1755 | 31.8 ± 14.51, 29.5 (21, 40.5) |  |
| CRP (mg/L) | N | 615 | 46.35 ± 60.39, 18.49 (5.7, 69.38) | <0.001 |
|  | Y | 829 | 71.1 ± 72.39, 49.93 (12, 102.42) |  |
| HS-CRP (mg/L) | N | 729 | 30.67 ± 51.28, 8.25 (2.83, 35.2) | <0.001 |
|  | Y | 1420 | 42.45 ± 63.87, 13.52 (4.03, 53.7) |  |

**Supplementary Table 3**: Distribution of Lab values in risk subgroups

| Variable | Risk Groups | N | Mean ± SD, median (interquartile) | Wilcoxon Rank sum test  p-value t |
| --- | --- | --- | --- | --- |
| CRP  mg/L | High | 183 | 65.26 +/- 69.61, 41.86 (8.97, 102.27) | 0.793 |
|  | Low | 255 | 66.6 +/- 72.15, 37.23 (9.74, 103) |  |
| HS-CRP  mg/L | High | 252 | 45.1 +/- 66.12, 14 (3.85, 66.17) | 0.013 |
|  | Low | 303 | 31.13 +/- 51.44, 9.71 (3.14, 32.5) |  |
| HDL  mg/dL | High | 377 | 40.06 +/- 15.15, 38 (30.5, 48.53) | 0.215 |
|  | Low | 527 | 41.76 +/- 16.79, 39.5 (30, 50.5) |  |
| LDL  mg/dL | High | 311 | 82.34 +/- 36.85, 78 (55.33, 100.67) | 0.543 |
|  | Low | 438 | 80.94 +/- 35.93, 75 (56, 100) |  |
| TRIG  mg/dL | High | 476 | 166.75 +/- 101.37, 144.53 (99.5, 203.16) | 0.517 |
|  | Low | 641 | 163.28 +/- 97.87, 140 (96, 211) |  |
| VLDL  mg/dL | High | 329 | 30.08 +/- 13.52, 29 (19, 38) | 0.073 |
|  | Low | 469 | 28.84 +/- 14.57, 25 (17, 39) |  |

**Supplementary Table 4**: Lab test characteristics stratified by the leukemia status for the low-risk group (n=1783)

| Variable | Leukemia | N | Mean ± SD, median (interquartile) | Wilcoxon Rank sum test  p-value |
| --- | --- | --- | --- | --- |
| CRP  mg/L | N | 127 | 52.67 +/- 69.04, 19.5 (7.93, 76.71) | 0.001 |
|  | Y | 128 | 80.42 +/- 72.77, 64.62 (13.69, 129.48) |  |
| HS-CRP  mg/L | N | 133 | 26.96 +/- 48.43, 8.59 (2.98, 29.96) | 0.226 |
|  | Y | 170 | 34.4 +/- 53.59, 10.44 (3.41, 36.1) |  |
| HDL  mg/dL | N | 234 | 42.89 +/- 16.13, 40.42 (31, 52) | 0.076 |
|  | Y | 293 | 40.85 +/- 17.28, 38.67 (29.5, 49) |  |
| LDL  mg/dL | N | 204 | 82.95 +/- 38.15, 79 (55.5, 101) | 0.392 |
|  | Y | 234 | 79.18 +/- 33.85, 72 (56, 99) |  |
| TRIG  mg/dL | N | 286 | 160.8 +/- 98.68, 139 (91.58, 208) | .395 |
|  | Y | 355 | 165.27 +/- 97.3, 140.33 (98.25, 213.77) |  |
| VLDL  mg/dL | N | 212 | 29.03 +/- 16.49, 23.63 (16, 41) | .312 |
|  | Y | 257 | 28.68 +/- 12.8, 26 (19, 38) |  |

**Supplementary Table 5**: Lab test characteristics stratified by the leukemia status for the high-risk group (n=1003)

| Variable | Leukemia | N | Mean ± SD, median (interquartile) | Wilcoxon Rank sum test  p-value |
| --- | --- | --- | --- | --- |
| CRP  mg/L | N | 82 | 46.08 +/- 55.75, 22.11 (3.63, 72.69) | <0.001 |
|  | Y | 101 | 80.83 +/- 75.85, 67.39 (13.6, 115.31) |  |
| HS-CRP  mg/L | N | 98 | 42.42 +/- 65.04, 14.4 (4.35, 60) | 0.765 |
|  | Y | 154 | 46.8 +/- 66.96, 14 (3.45, 70.03) |  |
| HDL  mg/dL | N | 135 | 40.09 +/- 15.64, 39 (30.5, 49.17) | 0.883 |
|  | Y | 242 | 40.05 +/- 14.9, 37.55 (30, 48) |  |
| LDL  mg/dL | N | 110 | 85.35 +/- 38.42, 83.7 (57, 107) | 0.271 |
|  | Y | 201 | 80.69 +/- 35.96, 76.5 (55, 98.8) |  |
| TRIG  mg/dL | N | 175 | 156.61 +/- 95, 136.33 (96, 180) | 0.046 |
|  | Y | 301 | 172.64 +/- 104.61, 148.5 (105, 217) |  |
| VLDL  mg/dL | N | 118 | 28.42 +/- 13.21, 26.25 (18.46, 33.5) | 0.069 |
|  | Y | 211 | 31.01 +/- 13.63, 29.67 (19.6, 39) |  |
